# Volatile Organic Compounds Obtained by *in Vitro* Callus Cultivation of *Plectranthus ornatus* Codd. (Lamiaceae)

**DOI:** 10.3390/molecules180910320

**Published:** 2013-08-26

**Authors:** Helna C. Passinho-Soares, Paloma R. Meira, Juceni P. David, Paulo R. R. Mesquita, Ademir E. do Vale, Frederico de M. Rodrigues, Pedro A. de P. Pereira, José Raniere F. de Santana, Fabio S. de Oliveira, Jailson B. de Andrade, Jorge M. David

**Affiliations:** 1 Faculdade de Farmácia, Universidade Federal da Bahia, Rua Barão de Jeremoabo, s/n, 41810-290, Salvador (BA), Brazil; E-Mails: helna@ufba.br (H.C.P.-S.); palomaribeiromeira@hotmail.com (P.R.M.); advale@gmail.com (A.E.V.); 2 Programa de Pós- graduação em Biotecnologia, Universidade Estadual de Feira de Santana, 44031-460, Feira de Santana (BA), Brazil; E-Mail: jose.raniere@gmail.com; 3 Instituto de Química, Universidade Federal da Bahia, Campus de Ondina, 40170290, Salvador (BA), Brazil; E-Mails: paulinho_rmesquita@hotmail.com (P.R.R.M.); pedroapp@ufba.br (P.A.P.P.); jailsong@ufba.br (J.B.A.); jmdavid@ufba.br (J.M.D.); 4 Empresa Baiana de Desenvolvimento Agrícola S.A, 40170-110, Salvador (BA), Brazil; E-Mail: fredericomr@hotmail.com; 5 Centro de Ciências da Saúde, Universidade Federal de Recôncavo Baiano, 44574-490, Santo Antonio de Jesus (BA), Brazil; E-Mail: fabioso@ufrb.edu.br

**Keywords:** *Plectranthus ornatus*, Lamiaceae, *in vitro* cultivation, HCA and PCA, HS-SPME, volatile compounds

## Abstract

*Plectranthus* spp (Lamiaceae) are plants of economic importance because they are sources of aromatic essential oils and are also cultivated and several species of this genus are used as folk medicines. This paper describes the effects of different concentrations of the 2,4-dichlorophenoxyacetic acid (2,4-D) and 1-naphthaleneacetic acid (NAA) on the induction of callus from nodal segments of *Plectranthus ornatus* Codd and in the production of volatile organic compounds (monoterpenes and sesquiterpenes). The 20 and 40 day calli were subjected to solid phase micro extraction (HS-SPME) and submitted to GCMS analysis. Variations in VOCs between the samples were observed and, a direct relationship was observed between of the major constituent detected (α-terpinyl acetate) and the monoterpenes α-thujene, α-pinene, β-pinene, camphene, sabinene and α-limonene that were present in the volatile fractions. Besides α-terpinyl acetate, isobornyl acetate and α-limonene were also major constituents. Variations were observed in VOCs in the analyzed periods. The best cultivation media for the production of VOCs was found to be MS0 (control). Moderate success was achieved by treatment with 2.68 µM and 5:37 µM NAA (Group 2). With 2,4-D (9.0 µM), only the presence of α-terpinyl acetate and isocumene were detected and, with 2.26 µM of 2,4-D was produced mainly α-terpinyl acetate, α-thujene and β-caryophyllene (16.2%). The VOC profiles present in *P. ornatus* were interpreted using PCA and HCA. The results permitted us to determine the best cultivation media for VOC production and, the PCA and HCA analysis allowed us to recognize four groups among the different treatments from the compounds identified in this set of treatments.

## 1. Introduction

*Plectranthus* is considered to be the largest genus of the Lamiaceae family, and is comprised of *ca.* 300 species and various hybrids. *Plectranthus* species are of economic importance because they are sources of aromatic essential oils and are also cultivated as ornamental plants, which are edible or are used as medicinal herbs as anthelmintics, antiseptics and purgatives, or to treat ear infections, intestinal spasms or nausea and vomiting [1]. However, there have only been a limited number of pharmacological and phytochemical studies on these plants [2].

*Plectranthus ornatus* Codd is an aromatic herb, known in Brazil as “boldo”, “boldo-de-jardim”, or “boldo-do-Brasil”. This species is used in folk medicine to treat liver failure and dyspepsia. Terpenoids are particularly abundant in several species of the Lamiaceae family and are considered to be the main factors responsible for the biological activities of these *Plectranthus* [3]. Studies on the chemical composition of the essential oils of the *Plectranthus* genus show that species in this genus are rich in monoterpenes and sesquiterpenes [1,4].

Essential oils can be obtained from wild or cultivated plants, but the harvest of large quantities of wild plants should be avoided. Thus, effective protocols for micropropagation of plants should therefore be developed so that the chemicals of interest can be extracted without the need for exploitation of wild populations [5,6]. There is a special interest in obtaining secondary metabolites *in vitro* since this media provides comprehensive control of production conditions, allowing the compounds of interest to be synthesized [7,8]. On the other hand, nowadays biotransformation by using plant cell cultures has been applied effectively for the production of bioactive derivatives [9]. In addition, the cultivation of callus and cells permit the elucidation of factors involved in secondary metabolism, which are necessary in order to produce *in vitro* compounds of medicinal importance [10].

In a range of auxins employed in callus production 2,4-D and NAA are the most frequently used. 2,4-D can induce very high frequencies of embryogenesis, and is routinely more efficient. However, 2,4-D usually develops embryos with abnormal morphology and slow to develop into plantlets. The frequency of embryogenesis and the mean number of embryos per culture is lower with NAA comparing with 2,4-D, however it exhibits more defined morphology. Therefore, both compounds in addition to MS0, are the auxins of choice for routine callus production [11].

This paper describes the volatile organic compounds obtained from calli derived *in vitro* from nodal segments of *P.* *ornatus*. These volatiles were collected by HS-SPME, a solvent-free sample preparation technique that has been employed in different studies dealing with volatile compound analysis [12]. A total of 19 compounds were identified, consisting mainly of monoterpenes and sesquiterpenes whose levels varied depending on the period and the culture medium. The VOC profile was compared to that of field-grown adult plants and of callus produced *in vitro*. The results were also evaluated using multivariate analysis techniques such as PCA and HCA as an approach to detect tendencies and relationships.

## 2. Results and Discussion

Table 1 presents the results of callogenesis in *P. ornatus* and the formation of shoots and roots is influenced by the equilibrium between the plant growth regulators BAP and NAA.

**Table 1:** Average values for the presence of callus at the explant base (EB) and the explant apex (EA) and the number of shoots (NS) and leaves (NL) in *P. ornatus* at different concentrations of BAP and NAA.

NAA (µM)	BAP (µM)					
	4.5		9.0		18.0	
	Original average	Processed average	Original average	Processed average	Original average	Processed average
	EB					
0.0	90.8	77.2aA *	100.0	90.0aA	100.0	90.0aA
5.4	100.0	90.0aA	96.4	84.2aA	87.5	78.7aA
10.1	100.0	90.0aA	100.0	90.0aA	100.0	90.0aA
21.5	100.0	90.0aA	93.7	82.5aA	100.0	90.0aA
	EA					
0.0	30.8	32.2abA	41.5	40.6aA	12.5	11.2bA
5.4	33.3	31.9abA	50.0	45.0aA	8.3	8.7bA
10.1	50.0	44.9aA	25.0	26.2aA	34.8	36.0aA
21.5	32.0	34.5aA	31.2	37.5aA	13.7	18.7aA
	NS					
0.0	1.4	1.5aAB	2.2	1.7aAB	1.8	1.6aA
5.4	1.3	1.5aB	3.1	2.0aA	1.3	1.5aA
10.1	3.1	1.9aA	1.0	1.4bB	0.9	1.3bB
21.5	0.8	1.3aB	1.2	1.4aB	0.9	1.3aB
	NL					
0.0	5.2	2.4bA	12.3	3.6aB	7.1	2.8bA
5.4	3.8	2.1bA	22.0	4.7aA	5.4	2.5bAB
10.1	6.8	2.7aA	3.9	2.2abC	2.7	1.8bBC
21.5	3.3	2.0aA	3.4	2.1aC	0.5	1.1aC

* Means followed by the same small letters in each line and by the same capital letters in each column are not significantly different (*p* < 0.05) using Tukey test.

No significant differences between the treatments on callus formation at the explant base were observed. We obtained up to 100% callus formation in explants in the presence of 9.0 µM or 18.0 µM BAP and no NAA, indicating that the addition of auxin is not necessary for the induction of callus at the base of *P. ornatus* explants. The formation of callus can be attributed to the action of endogenous auxin accumulated on the cutting edge of the baseline, which stimulates cell proliferation, especially in media enriched with cytokinins [13].

However, the interaction between the treatments with respect to callus genesis at the apex of explants was significant. For the apical callus genesis, no difference was observed between different concentrations of NAA. However, in media without or with 5.4 µM NAA, the best results were obtained using 9.0 µM BAP. The results differed notably with 18.0 µM BAP, indicating that the auxin/cytokinin pro-auxin equilibrium is advantageous to the formation of callus at the apex of *P. ornatus* explants. 

Interactions for the variables, number of shoots per explant and number of leaves per shoot were observed between treatments. Averages of 0.9 to 2.0 shoots of *P. ornatus* were obtained in these treatments. The highest averages for shoots and leaves were obtained in media supplemented with 9.0 µM BAP in the absence or presence of 5.4 µM NAA.

On the other hand, the callogenesis of *P. ornatus* demonstrated that the callus growth, aspect and consistency differ according to the type and concentration of auxin employed. The nodal explants were found to produce more developed callus when cultured in a medium containing 2.68 and 5.37 µM of NAA and 2.26 µM of 2,4-D.

**Figure 1:**
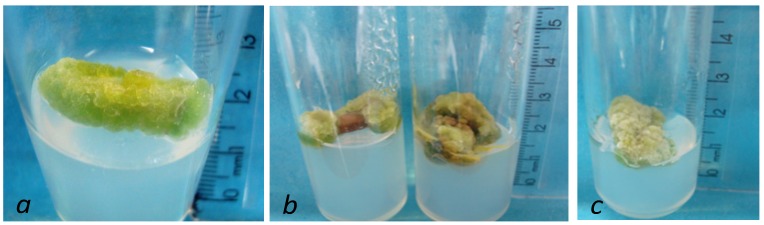
Morphological aspects of callus of *P. ornatus* employing (**a**) NAA, (**b**) 2,4-D and (**c**) MS0.

The explants cultured on NAA showed a good formation of callus with green and hard aspect, while those cultured in a medium supplemented with (2,4-D) showed callus that was green and hard in the center and white and friable on the ends. On the other hand, the callus from explants cultured in media without supplementation of regulators (MS0) had green and friable features (Figure 1). 

**Figure 2:**
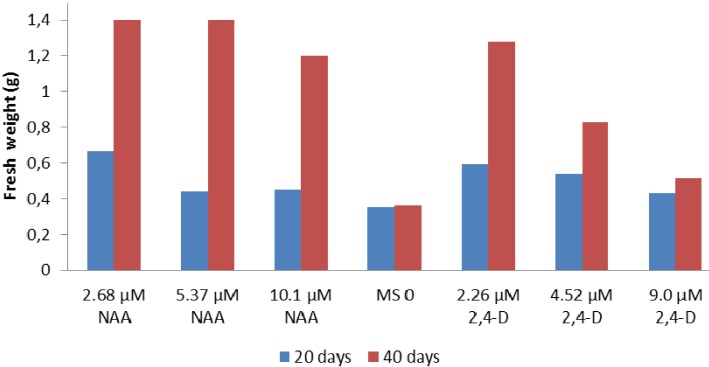
Fresh weight de calli using diferent regulators and inoculation time.

The biomass gain in callus was found to occur between 20 and 40 days after inoculation, except for the callus cultured in a medium supplemented with 9.0 µM of 2,4-D or in an MS0 medium (Figure 2). It is noteworthy that after 30 days of inoculation with MS0, the medium began a stage of decline. After this time, the medium demonstrated a reduced regenerative ability, showing callus with a brown color in which VOC HS-MEFS-GC/MS analyses were not possible. The main yield of fresh material obtained was achieved with treatments carried out with 2.68 and 5.37 μM of NAA and 40 days of inoculation. 

The components of the volatile oils of *P. ornatus* obtained from callus cultured *in vitro* with different concentrations of the auxin NAA and 2,4-D, and with 20 and 40 days of inoculation were collected and identified by HS-SPME-GC/MS and the relative area % are described in Table 2. A total of 19 compounds were found, consisting mainly of monoterpenes and sesquiterpenes (all constituents with relative peak areas are higher than 0.5% were considered).

**Table 2:** HS-SPME area % of VOCs of the *in vitro* calli cultures of *P. ornatus* ^1^.

Peak no.	Compound	RI_exp_	RI_lit_	MS0a	NAA (µM)						2,4-D (µM)				
					2.68a	5.37a	10.1a	2.68b	5.37b	10.1b	2.26a	4.52a	9.0a	2.26b	4.52b
1.	α-thujene	917	923	-	-	-	1.8	-	0.2	-	-	-	-	-	-
2.	α-pinene	926	933	9.0	1.9	3.1	5.4	1.6	2.4	2.2	6.9	-	-	2.2	-
3.	camphene	941	952	8.0	1.8	2.9	5.2	1.3	2.3	0.9	-	2.0	1.6	2.1	-
4.	sabinene	972	973	2.8	1.9	1.7	-	0.8	1.3	2.1	-	2.1	-	-	-
5.	β-pinene	977	980	2.1	2.5	3.9	6.4	2.1	2.9	2.9	-	-	-	2.7	-
6.	1-octen-3-ol	976	978	-	-	-	6.0	-	-	-	-	2.6	-	-	-
7.	α-limonene	1,033	1,031	16.5	5.6	12.8	9.8	4.0	6.2	2.9	-	6.8	5.4	5.3	-
8.	terpinolene	1,087	1,084	-	-	-	-	-	0.6	1.4	-	-	-	-	-
9.	borneol	1,133	1,165	2.9	1.4	3.6	5.7	1.0	1.8	-	-	2.0	-	3.0	-
10.	*(Z)-p*-menth-1-ol	1,135	1,136	-	0.4	1.1	-	-	0.5	-	-	0.5	-	2.0	-
11.	*p*-menth-8-ol	1,141	1,144	0.7	0.7	1.2	-	0.6	0.6	-	-	-	-	-	-
12.	isobornyl acetate	1,287	1,285	16.8	15.6	2.7	11.3	16.4	19.2	21.4	-	1.1	-	-	-
13.	α-terpinyl acetate	1,356	1,350	31.3	46.4	51.6	35.9	60.4	56.3	63.0	72.1	73.5	73.6	57.7	53.4
14.	β-isocumene	1,408	1,403	4.0	1.7	2.8	3.2	0.7	2.0	2.1	-	2.0	1.6	3.1	1.5
15.	decanol acetate	1,412	1,409	1.8	1.6	-	-	-	-	2.5	-	-	-	-	-
16.	β-caryophyllene	1,420	1,418	1.5	0.7	1.0	-	0.4	0.3	-	15.2	-	-	-	-
17.	α-humulene	1,454	1,454	3.3	1.9	1.8	3.0	1.7	-	-	-	1.8	-	-	-
18.	kessane	1,532	1,528	-	-	-	-	-	7.8	3.1	-	-	-	-	-
19.	sesquisabinene hydrate	1,543	1,545	1.5	1.2	1.8	3.6	1.2	1.7	2.4	-	1.5	-	1.8	-

^1^ RI, Retention index, MS0, MS media not containing supplementation of regulators; a, 20 days after inoculation; b, 40 days after inoculation.

**Figure 3:**
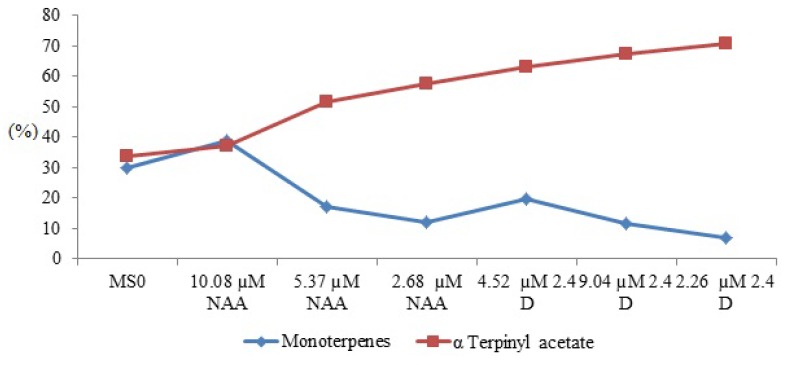
Correlation between quantities of α-terpinyl acetate and monoterpenes present in VOCs of *P. ornatus*.

The results also indicated qualitative and quantitative variations of constituents in the two periods analyzed. They showed a remarkable difference between VOCs composition from the mother plant and *in vitro* callus, differences of components from the callus obtained with NAA, 2,4-D and MS0 and a direct correlation between the percentage of α-terpenyl acetate and the other monoterpenes in the callus of 20 and 40 days of inoculation (Figure 3). The presence of terpinolene and kessane was only qualitatively detected in the callus grown for 40 days (Table 2), whereas the hydrocarbon 1-octen-3-ol was seen in the callus grown for 20 days. 

The callus grown for 40 days showed a decrease in β-pinene, α-limonene, isocumene and β-caryophyllene, as well as an increased production of isobornyl acetate in the callus cultured in a medium supplemented with NAA (Table 2). The isobornyl (area > 10%) was found to be present only in volatile fractions obtained from callus cultured in medium containing the auxin NAA for 20 and 40 days, and in the control medium (MS0) cultured for 20 days. The overproduction of β-caryophyllene was also demonstrated in the callus cultured for 20 days in a medium supplemented with 2.26 μM 2,4-D in comparison with the callus cultured with NAA (2.68 μM and 5:37 μM) in the same period (Table 2). 

Comparing the volatile compounds of 20 day old callus cultured on MS0 (control) with those cultured on an MS medium supplemented with different concentrations of the auxins showed that the addition of 10.08 μM of NAA and 4.52 μM of 2,4D resulted in a significant increase in the levels of β-pinene and decrease of α-limonene (comparing with MS0). Similar results were found [14] regarding the influence of auxin and cytokinin on the production of essential oils in *Lantana camara* L. 

In the cultivated medium without the regulator (MS0) increases of HS-SPME areas of α-pinene (9.0%), camphene (8.0%), sabinene (2.8%), and α-humulene (3.3%) were observed, while the relative presence of α-terpinyl acetate (31.3%) decreased. Variations were also observed in α-thujene and indicated the presence of this compound in 20 day old edge callus cultured in a medium supplemented with 10 μM of NAA and 2.26 μM of 2,4-D. Traces of this compound (0.2%) were also observed in calli cultured in a medium supplemented with 5.37 μM of ANA for 40 days. Thus, this study demonstrated considerable variations in the VOCs. The VOCs are influenced by cultivation time, the specific regulators and the concentrations of the regulators that are used in the preparation of culture media. Similar results in terms of the qualitative and quantitative variation of VOCs in *in vitro* studies were observed in *Lycopersicon esculentum* Mill [15], *Thymus vulgaris* [16] and *Olea europaea* [17]. 

The analysis of VOCs present in the callus of *P. ornatus* indicated that the decrease in the proportions of α-terpinyl acetate in the volatiles was equal to the increase of monoterpenes in the samples, as shown in Figure 3. In this case, the relative overproduction of α-terpinyl acetate composition in the volatile oil from callus and the low concentrations of other monoterpenes in the VOCs seem to be directly associated, since the α-terpinyl cation is the precursor of many of these compounds. This result confirms those previously reported in the literature [18,19]. VOCs obtained *in vitro* were characterized by a high percentage of monoterpenes (52.2%). 

Comparable results for the production of VOCs under regulator influence with a high production of monoterpenes was obtained for *Melissa officinalis* L. [20] and *Salvia officinalis* L. [21]. Table 3 describes the 33 VOCs identified in the mother plant (the matrix plant). Comparing the results from Table 1 and Table 2 shows that there are 12 similar compounds both in the *in vitro* and *ex vitro* plants, 8 of which are monoterpenes.

**Table 3:** HS-SPME area % of VOCs observed in mother plants (*ex vitro*) of *P. ornatus.*

Peak no.	Compound	RI_exp_	RI_lit_	Relative area (%)
1.	2-hexenal	854	854	0.50
2.	α-thujene	917	921	13.64
3.	α-pinene	926	939	9.97
4.	sabinene	972	976	5.46
5.	β-pinene	977	980	3.09
6.	1-octen-3-ol	976	978	9.23
7.	β-myrcene	992	991	0.40
8.	3-octanol	995	993	0.81
9.	(*Z*)-hexenol acetate	1,005	1,004	0.16
10.	α-limonene	1,033	1,031	0.43
11.	(*Z*)-β-ocimene	1,038	1,040	0.21
12.	(*E*)-β-ocimene	1,048	1,050	2.54
13.	Decanal	1,142	1,204	0.10
14.	α-cubebene	1,356	1,351	0.69
15.	α-copaene	1,381	1,376	1.59
16.	β-bourbonen	1,389	1,384	0.88
17.	β-cubebene	1,392	1,390	1.35
18.	α-gurjunene	1,397	1,409	1.75
19.	β-caryophyllene	1,420	1,418	30.34
20.	β-gurjunene	1,429	1,432	0.41
21.	(*Z*)-β-farnesene	1,434	1,433	0.15
22.	(*Z*)-muurola-4(14),5-diene	1,444	1,460	0.14
23.	(*E*)-β-farnesene	1,454	1,458	0.18
24.	α-humulene	1,454	1,454	1.82
25.	alloaromadendrene	1,468	1,461	0.19
26.	γ-muurolene	1,471	1,477	0.08
27.	germacrene D	1,479	1,480	8.20
28.	β-selinene	1,489	1,485	0.25
29.	Β-guaiene	1,494	1,490	0.29
30.	(*E*)-β-guaiene	1,498	1,500	3.77
31.	germacrene-D-ol	1,586	1,574	0.58
32.	caryophyllenoxide	1,592	1,581	0.72
33.	α-muurolol <epi>	1,641	1,641	0.08

**Figure 4:**
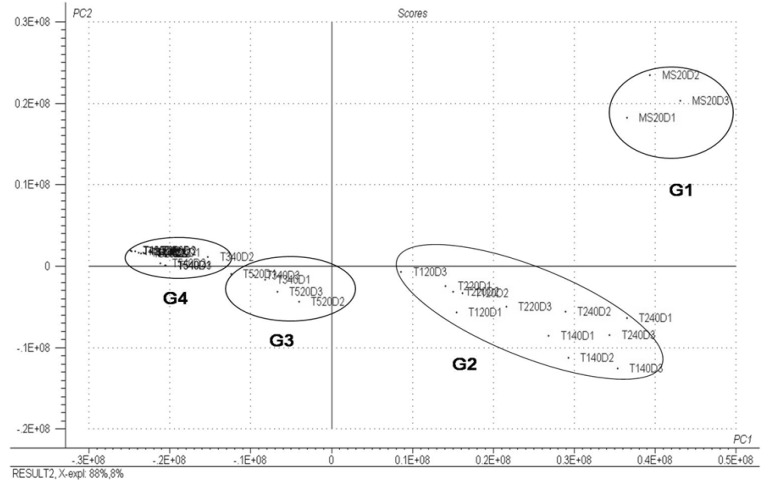
PCA scores for PC1 × PC2; each point represents a sample of valuated oil.

Figure 4 shows the scores chart obtained from a PCA. Each point in this figure represents one of the samples of volatile fraction evaluated. This figure indicated that it was possible to detect similarities between the replicas of the three calli in the MS0 medium within 20 days. MS20D samples were grouped together in the analysis (termed Group 1) and were distinct from other samples. Another group identified, Group 2, included the treatments with 2.68 µM NAA, 5.37 µM NAA at 20 days, and 2.68 µM treatments 5:37 µM NAA and NAA at 40 days, which were referred to as T120D, T220D, T140D and T240D, respectively. Group 3 was composed of treatments with 4:52 µM of 2,4D, 10.1 µM NAA at 20 days and 10.1 µM NAA with 40 days, codified as T520, T320D and T340D, respectively. The last group, identified as Group 4, was composed of treatments with 2,4-D at concentrations of 2.26 µM of, 9.0 µM at 20 days, and with 2.26 µM and 4.52 µM at 40 days, respectively referred to as T420D, T620D, and T440D T540D. Group 4 showed the greatest similarity between the chromatographic profiles in the sample treatments.

The results indicate that the best medium for the production of VOCs was MS0 (control) followed by treatment with 2.68 µM and 5.37 µM NAA (Group 2). The media resulting in the lowest production of VOCs occurred with 2,4-D at a concentration of 9.0 µM and 2.26 µM. Only α-terpinyl acetate and isocumene, as well as α-terpinyl acetate, α-thujene and β-caryophyllene, respectively, were detected in these media.

**Figure 5:**
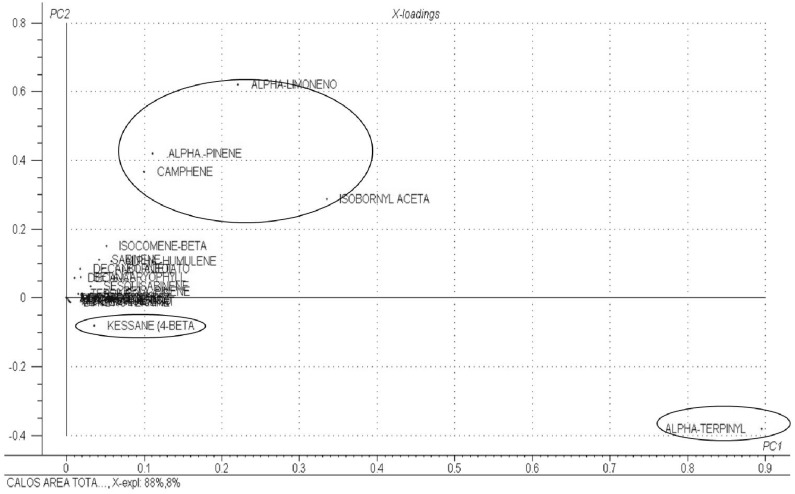
Graph of PCA loadings for PC1 × PC2.

Figure 5 represents the loadings obtained by PCA. The VOCs α-pinene, α-limonene, camphene and isobornyl acetate showed similar area values between treatments. Kessane and α-terpinyl acetate also showed significant loading values in this figure, which did not show similar behavior. The other COVs present had similarly low loading values, indicating that these compounds did not contribute to the variability of the data set, with approximately constant values around the average. 

Evaluation of the two charts represented in Figure 3 and Figure 4 demonstrated that treatments in Group 1 showed higher values of α-pinene, α-limonene, camphene and isobornyl acetate, while treatments in Group 2 showed higher values of α-terpinyl acetate and, to a lesser extent, of kessane, this occurred only in 40 day old edge callus cultured in a medium supplemented with 5:37 µM NAA. The treatments with higher levels of α-terpinyl were treatments T140D and T220D (2.68 µM and 5:37 µM NAA).

These results show the importance of growth regulators on the induction of callogenesis and on the production of VOCs at calli. Where nodal explants produced more developed calli when cultured in a medium containing 2.68 µM and 5:37 µM of NAA and 2.26 µM of 2,4-D. The PCA analysis identified similarities of the volatile compounds among the different treatments.

Since the culture conditions used in this work, such as the amount of light, temperature, medium composition, pH and age of callus, were completely controlled, the results showed that the profile of the VOCs differed only according to the type and concentration of auxin used. 

All of these groups were also observed by the PCA (Figure 4 and Figure 5). This indicates that the profile of the VOCs is directly related to the type and concentration of the culture medium regulators. These data are consistent with those in the literature [20] indicating the influence of growth regulators on the essential oil composition of *Melissa officinalis* L.

The *P. ornatus* calli cultured on MS0 (Table 2) showed the main presence of α-pinene, α-limonene, camphene, isobornyl acetate and, humulene. These results suggest that the addition of regulators into the culture medium interferes with the synthesis of these compounds.

**Figure 6:**
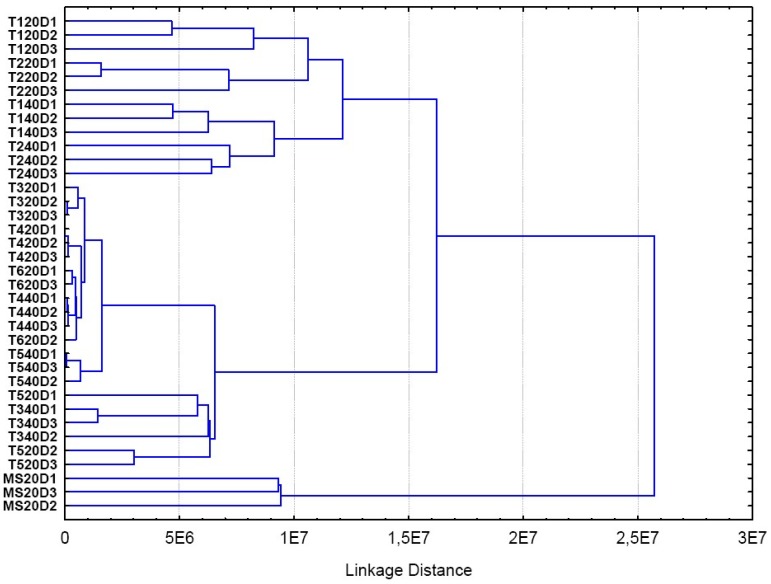
HCA dendrogram.

The HCA analysis confirmed that the same treatment groups obtained by PCA are also evident using a dendrogram (Figure 6). The same groups were identified among the samples evaluated using two different chemometric techniques. Thus, these results corroborate many of the conclusions above described.

## 3. Experimental

### 3.1. Plant Material

The explants were removed from the mother (matrices) plants and were grown at the Faculdade de Farmácia da Universidade Federal da Bahia (UFBA). A voucher has been deposited at the Herbarium do Museu Nacional da Universidade Federal do Rio de Janeiro under the number R196538.

### 3.2. *In Vitro* Establishment of *Plectranthus Ornatus*

The *in vitro* culture of *P. ornatus* was established according the following protocol; the explants were disinfested by washing the leaves in running water for 60 min. Then, in a laminar flow chamber, the leaves were immersed in ethanol 70% (v/v) for 1 min followed by sodium hypochlorite (2.0% of active chlorine) and 3 drops of neutral detergent for 15 min. Then the segments were washed three times in sterile distilled water. The 1.5 cm long explants were individually inoculated in test tubes containing MS medium (10 mL) [21], supplemented with 3% sucrose, 0.6% agar and different combinations of BAP (0, 4.5, 9.0, and 18.0 µM) and NAA (0, 5.37, 10.08 and 21.48 μM). The pH of the culture medium was adjusted to 5.7 ± 0.1, using KOH or 0.1 N HCl, before autoclaving. The cultures were kept in a growth room at 25 ± 3 °C with a photoperiod of 16 h, 60% relative humidity and 30 µmol. m^−2^ s^−1^ of photosynthetically active radiation. Sixty days after inoculation, the explants were evaluated for the presence of callus at the apex and base, the number of shoots and the number of leaves. The experimental design employed was completely randomized in a 3 × 4 (BAP × NAA) factorial arrangement with four replicates *per* treatment, each replicate consisting of 10 tubes. The rooted shoots were planted in disposable cups containing a 2:1:1 mixture of autoclaved soil, sand and vermiculite. The plants were incubated in growth chambers at 25 ± 2 °C and 100% humidity. The humidity was gradually eliminated after 10 d, by which time the plants had produced one pair of new leaves. After 3 weeks, the plants were transferred to a garden soil mixture and maintained in a greenhouse. The data were evaluated by the Tukey test and, percentages were transformed into the arcsine 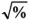 and the counting numbers on 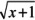. Data were analyzed using the SISVAR program, v 4.3, developed by Ferreira [22].

### 3.3. *In Vitro* Production and Establishment of Callus Tissues

The production of callus of *P. ornatus* was induced from nodal segments which were cultivated in a solidified medium as described by Murashige and Skoog [23] employing 6 g.L^−1^ of agar, 87.64 mM sucrose supplemented with NAA (2.68; 5.37 and 10.08 μM) and 4.5 μM of BAP; 2.4-D (2.26; 4.52 and 9.04 μM) and 4.5 μM BAP; and, as a control, explants inoculated in MS media without a regulator (MS0). The explants were disinfected by washing in running water for 40 min and were sequentially rinsed twice with distillated water. The explants were immersed in an aqueous ethanol solution (70%) in an aseptic chamber for 1 min with constant agitation and were then left in the stand for 15 min in a NaOCl solution (2%) with Tween 20 (20 drops/L). The explants were then rinsed with autoclaved distilled water for approximately 3 min. After disinfection, the 1.5 cm explants were inoculated individually in test tubes (2.5 × 15 cm) containing 12 mL of culture medium. The test tubes containing the medium and the explants were sealed with plastic caps without plastic wrap, kept in the dark for eight days and then submitted to a photoperiod of 16 h of cool white light under a photon flux density of 25 μmol.m^−2^.s^−1^. Calli characteristics such as consistency, texture, color, and the profile of VOCs were evaluated. The tubes containing contamination were removed from the experiment and evaluations were performed after 20 and 40 days of growth 

### 3.4. Characterization of Metabolites Produced in Callus Tissues

#### Extraction of Volatile Organic Compounds (VOC)

The VOCs were extracted employing headspace and solid phase micro extraction [24] and were analyzed by GCMS. All extractions were carried out in triplicate with 1.0 g of *in vitro* cultivated callus after 20 and 40 days of growth and, all the analysis were carried out on a maximum five consecutive days. Briefly, the callus were mashed in a glass vial (12 mL), covered up with an aluminum foil lid and silicone septa and, the sample was kept in repose for 20 min at room temperature. A holder needle with a 100 μm fiber coated polydimethylsiloxane (PDMS) mounted in a syringe-like (Supelco, Bellefonte, PA, USA) was then inserted into the sample vial which was heated on a heating plate at 60 °C during 20 min. The volatiles were thermally desorbed in the GC injector for 3 min at 260 °C. 

The analyses were performed in GC-MS equipment [CG-2010-QP-2010, Shimadzu) with the following conditions: Elite-5 MS Perkin Elmer column (30 m × 0.25 mm; 1 *μ*m film thickness) with a fused silica capillary column; temperature programmed to increase from 50 °C to 80 °C (1.5 °C min^−1^); temperature increases from 15 °C min^−1^ to 160 °C over a four minute period and 20 °C min^−1^ to 250 °C over a seven minute period (total time 40.5 min)]; a transfer line of 250 °C; helium, used as a carrier gas, was adjusted to a linear velocity of 40 cm s^−1^ (measured at 100 °C) with column flow of 1.22 mL.min^−1^ the split flow was adjusted to give a ratio of 30:1. The mass detector conditions were: transfer line and ion source temperatures of 250 °C; ionization mode with electron impact at 70 eV. 

The individual VOCs peaks were identified by comparison of the retention indices (RI), which were calculated for all volatile constituents using a homologous series of *n*-alkanes C_8_–C_32_ and the mass spectra using the same conditions [25]. The identification was also based on computer matching of the mass spectra NIST 147 Database. The percentage of individual peaks was achieved by peak area normalization measured without correction factors. 

Three replicates were done for each different medium, and the relative standard deviation (RSD) was calculated for compounds exceeding 1%. The evaluation of volatile organic compounds present in the calli of *P. ornatus*, even that with small peak areas in these types of samples are multivariate data and therefore were interpreted using PCA and HCA, which are chemometric multivariate methods to identify similarities and tendency of groups of compounds within the different treatments. Thus, a set of data obtained from the analysis of callus extracts of *P. ornatus* on the chromatographic peak area of 74 volatile organic compounds measured in triplicate for 12 different samples was used to construct a 36 × 74 data matrix. Thus, their significance in each treatment was evaluated using a PCA and HCA employing the statistical programs Statistica 7.0 (Statistic Inc., Tulsa, OK, USA) and Unscrambler 8.0 (CAMO, Woodbridge, NJ, USA).

## 4. Conclusions

The present work demonstrated the *in vitro* establishment of *Plectranthus ornatus* is influenced by the equilibrium between the plant growth regulators BAP and NAA. Besides, the results obtained permitted us to determine the best cultivation media for VOC production from callus. The amount of light, temperature, medium composition, pH and age of callus, were completely controlled, the results showed that the profile of the VOCs differed only according to the type and concentration of auxin used. The PCA and HCA analysis helped in recognizing four groups among the different treatments from the compounds identified in this set of treatments. They indicated that the profile of the VOCs is directly related to the type and concentration of the culture medium regulators used.
